# The role of scene type and priming in the processing and selection of a spatial frame of reference

**DOI:** 10.3389/fpsyg.2013.00182

**Published:** 2013-04-10

**Authors:** Katrin Johannsen, Jan P. De Ruiter

**Affiliations:** SFB 673 Alignment in Communication, Fakultät für Linguistik und Literaturwissenschaft, Universität BielefeldBielefeld, Germany

**Keywords:** spatial perception, priming, psycholinguistics, cognitive processes, spatial reference frames, scene perception

## Abstract

The selection and processing of a spatial frame of reference (FOR) in interpreting verbal scene descriptions is of great interest to psycholinguistics. In this study, we focus on the choice between the relative and the intrinsic FOR, addressing two questions: (a) does the presence or absence of a background in the scene influence the selection of a FOR, and (b) what is the effect of a previously selected FOR on the subsequent processing of a different FOR. Our results show that if a scene includes a realistic background, this will make the selection of the relative FOR more likely. We attribute this effect to the facilitation of mental simulation, which enhances the relation between the viewer and the objects. With respect to the response accuracy, we found both a higher (with the same FOR) and a lower accuracy (with a different FOR), while for the response latencies, we only found a delay effect with a different FOR.

## Introduction

Expressing spatial relations is an important aspect of everyday communication. By using spatial terms, we indicate the location of one object in relation to another, to ourselves, to an interlocutor or to cardinal points. These different ways of expressing a spatial relation depend on the choice of frame of reference (FOR). A FOR can generally be described as a set of axes that defines space (Carlson, 1999). The point of intersection constitutes the origin (Miller and Johnson-Laird, 1976). The relative FOR establishes a ternary relationship which comprises a reference object, a located object, and a viewpoint. Using the intrinsic FOR, however, leads to a viewpoint-independent binary relationship between a reference object and located object (Levinson, 1996, 2003). In the present study, the origin of the relative FOR coincides with the egocentric perspective of the viewer whereas the origin of the intrinsic FOR is object-centered. The absolute FOR depends on environmental features such as cardinal points and will not be considered in the present study.

Crucially, spatial projective terms such as “next to,” “in front of” and “behind” (“neben,” “vor” and “hinter” in German) are ambiguous if it is unclear which FOR is adopted. Different FORs appear to be used differently in everyday life. There have been many attempts to identify preferences for specific FORs leading to ambiguous results. The relative FOR, being perceptually available and avoiding the extra computational effort needed for mental rotation, has been considered predominant by some authors (Linde and Labov, 1975; Levelt, 1982, 1989) whereas other authors have claimed that the intrinsic FOR predominates (Miller and Johnson-Laird, 1976) or is at least preferred (Carlson-Radvansky and Irwin, 1993; Carlson-Radvansky and Radvansky, 1996; Taylor et al., 1999). How FORs are chosen and maintained has been studied intensively (e.g., Carlson-Radvansky and Irwin, 1994; Carlson, 1999; Watson et al., 2004; Ball et al., 2009). Research has shown that, when choosing a FOR, all FORs are initially active (Carlson-Radvansky and Irwin, 1994) until one is selected. This selection is affected by various situational factors, for instance by functional relations between the objects (Carlson-Radvansky and Radvansky, 1996), motion characteristics (Levelt, 1984), gravity (Friederici and Levelt, 1990), or by alignment to the FOR chosen by the interlocutor in dialogue (Watson et al., 2004). These results indicate that there may not be a uniform default FOR but rather that the FOR selection is affected by situational influences.

A question that has, to our knowledge, not been addressed yet is whether the type of scene used to present the stimuli has a direct effect on the acceptability and processing of different FORs. This question arises from considerations of the disparities between FORs and of the role of embodiment.

A principle difference between the relative and the intrinsic FOR is that only the former requires the viewer as an origin. The relative FOR is indispensable for our navigation in the world as its use involves computation of relevant spatial relations. As the relative FOR originates in the viewer, an embodiment of the viewer may be considered a necessary prerequisite. Using the relative FOR for depictions therefore requires a mental simulation of a positioned viewer in the scene. Mental simulation in the processing of spatial relations irrespective of FOR has been reported elsewhere (e.g., Coventry et al., 2010).

We assume that there is little incentive for such a mental simulation of a viewer in depictions that exclusively involve configurations of objects and do not contain natural elements and that the relative FOR may thus be less preferred than the intrinsic FOR. However, if object configurations are embedded in depictions of natural environments, the use of the relative FOR may become more likely, as it is easier for a viewer to imagine being in a natural environment than in a scene that only contains “floating” objects.

Recent studies varied in their construction of scenes. Studies that have found a preference for the intrinsic compared to the relative FOR vary from using only a depiction of two or more objects without background elements (e.g., Carlson-Radvansky and Radvansky, 1996; Taylor and Rapp, 2004) to line-drawing scenes with rudimentary background elements (Carlson-Radvansky and Irwin, 1993). However, Taylor and Tversky (1996) showed that speakers chose different frames of reference for their descriptions of spatial environments depending on the characteristics of the scene they were shown.

We assume that presenting a realistic scene might result in a processing advantage for the relative FOR, as viewers are more likely to perform a mental simulation and establish a relation between the objects and themselves. Therefore, we hypothesize a higher acceptability of the relative FOR in more naturalistic scenes.

We investigated this hypothesis by presenting identical object configurations in two different scenes, and measuring the acceptability and reaction times (RTs) in a sentence-picture verification task. Sentences in German such as “Die Pflanze ist vor dem Stuhl” (“The plant is in front of the chair”) were used to assign a reference frame to the picture. In order to present a realistic scene, we chose a living-room scenario so that, in one version, the depiction showed a room with two embedded objects, whereas in the other version the same two objects were depicted in front of a white background.

In addition to the influence of scene type on FOR selection we were also interested in FOR-related priming effects. Recent studies have shown that the time needed for spatial term assignment in a FOR is prolonged when a different FOR has previously been processed (Carlson-Radvansky and Jiang, 1998; Carlson and van Deman, 2008). This effect^1^ has been interpreted as inhibition of the non-selected FOR (Carlson and van Deman, 2008). However, this investigation of priming effects focused on RTs, and did not include an analysis of response accuracy. If RTs were prolonged due to inhibition, we hypothesize that the accuracy ratings could also be affected. Inhibition of the non-selected FOR should lead to more rejections of targets following a prime trial with a different FOR than with a neutral or identical FOR. More rejections are expected because the inhibited FOR may not only be more difficult to process, which is revealed by longer response latencies, but also, in cases of stronger inhibition, be less available.

Even though priming effects in FOR selection have not been reported for RTs, other studies have suggested their possible existence. Watson et al. (2004) reported that interlocutors in dialog tended to use the same FOR as their interlocutor had previously used. This alignment effect was argued to result from priming of FORs. Thus, it is plausible to assume that not only can a different FOR delay responses, but that the same FOR can also speed up responses due to FOR priming, and that these effects might be observable in both RTs and accuracy ratings.

In order to assess these different possible effects of same and different FORs on accuracy and RTs, we included three conditions in the experiment: a match condition in which prime and target used the same FOR, a mismatch condition, which required switching the FOR between prime and target and a control condition, in which the prime trial did not disambiguate between FORs. Thus, the FOR on the target trial could either have been activated by having been used in the preceding trial (match condition) or inhibited by being available but not being selected (mismatch condition). The control condition served as a baseline for comparisons.

Our expectations were that having the same FOR would result in more acceptances of target trials in the match condition than in the control condition, while having different FORs would result in more rejected target trials in the mismatch condition than in the control condition. With regard to RTs, different FORs are predicted to lead to longer response latencies in mismatch target trials than in the control target trials, as described in earlier studies (Carlson-Radvansky and Jiang, 1998; Carlson and van Deman, 2008), while having the same FOR was predicted to yield shorter response latencies for target trials in the match condition compared to the control condition.

## Materials and methods

### Participants

Fifty students of Bielefeld University (21 men, 29 women) ranging in age from 20 to 62 (*M* = 26.9, *SD* = 8.5) were paid for their participation in the experiment. All participants were native German-speakers and each of them saw only one version (either the one with or the one without background).

### Stimuli and design

The experiment comprised 400 trials, consisting of 96 prime and 96 target trials as well as 200 distractor trials and 4 prime-target pairs with definite “no” answers according to both FORs. Sentence-picture verification for the prime trials were correct for the neutral configurations and for the relative and intrinsic FOR in 32 cases each. With regard to the target trials, 48 cases were correct for the relative and 48 for the intrinsic FOR. We thus made sure that 50% of the trials had as correct response “yes” and the other 50% had “no” as a correct response. The distractor trials consisted of 100 “yes” and 100 “no” response trials.

Stimuli consisted of a sentence and a picture presented subsequently. The sentence was presented auditorily (in German) and spatially described the object configuration in the picture. Sentence duration was approximately 2 s and during its presentation, participants saw a white screen. Immediately after the presentation of the stimulus sentence, the picture was shown.

**Figure 1:**
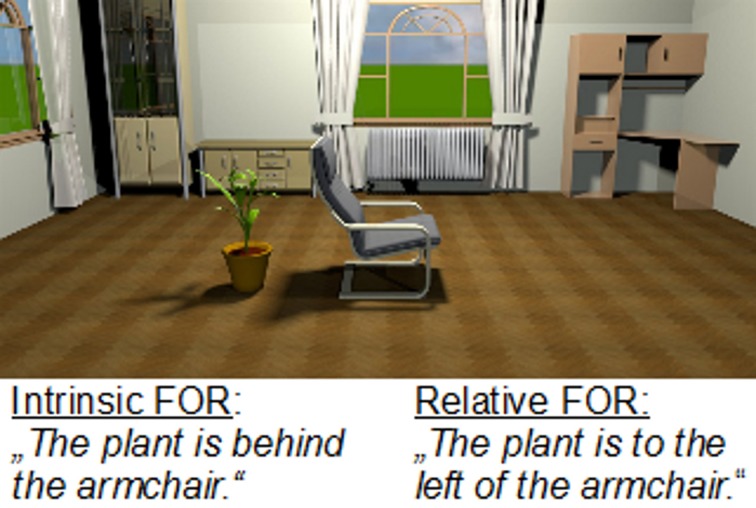
**Scene with background and both FOR**.

**Figure 2:**
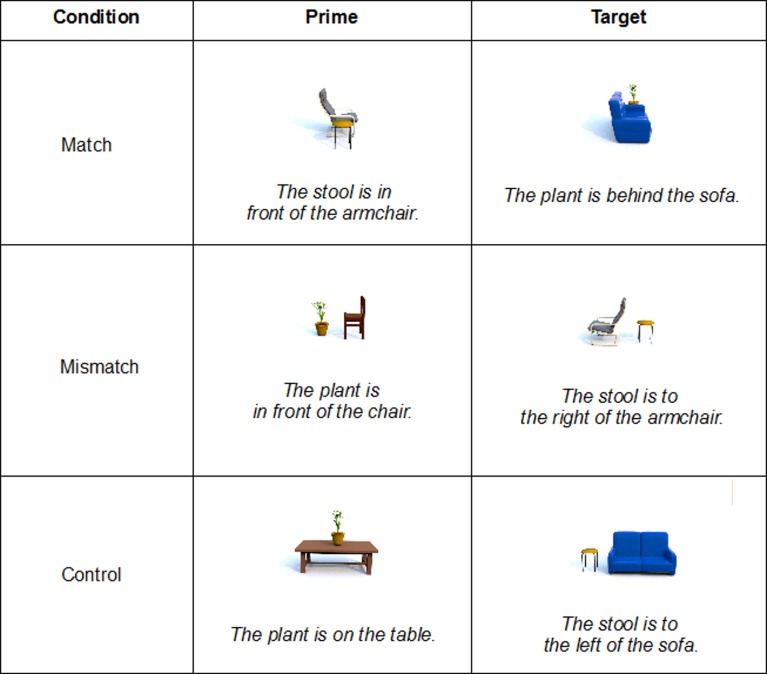
**Examples for prime-target-pairs in each condition**.

Pictures of object configurations were created using indoor planning software (“Sweet Home 3D”) in two versions which differed in background. In one version, following Henderson and Hollingworth's (1999) idea, we used a semantically coherent, human-scaled view of a living-room (Figure 1). This version constituted a *true scene* (Henderson and Ferreira, 2004), which will be referred to here as “scene with background.” In the other version, the object configurations were shown in front of a white background (Figure 2). This version (*ersatz scene*, Henderson and Ferreira, 2004) will be referred to here as “scene without background.” Participants saw either the version with or the version without background, therefore there were equal number of scenes with and without background. The size of the scene was 33 × 17 cm and the unconstrained viewing distance was approximately 70 cm.

Three types of pictures were created: experimental, neutral, and distractor. The experimental pictures consisted of two discrete objects in the foreground (reference object and located object). We used three different triaxial reference objects (chair, armchair, sofa), which were rotated on the vertical axis at angles of 0, 90, and 270° in order to vary the mapping of the horizontal intrinsic axes to the horizontal relative axes. Reference objects in the prime and target pictures had the same orientation and were always in an upright position. The located objects were biaxial (plant, stool) thus revealing no predefined horizontal orientation and were placed along the horizontal axes of the reference object (in front of, behind, to the left/right of). For the 0° rotation, the located object was only positioned to the left/right of in order to dissociate the relative and the intrinsic FOR. In order to keep the number of trials within a reasonable limit, we did not present every object in every possible combination of rotation, located object, and reference frame in all three conditions as this would have led to 180 prime target pairs. Thus, we reduced the number of target pictures to 16 which were presented with both FOR in all three conditions resulting in 96 prime target pairs. The 32 target trials (16 with a relative FOR and 16 with an intrinsic FOR) in each experimental condition consisted of 8 trials with 0° rotation, 12 trials with 90° rotation, and 12 trials with 270° rotation. The position of the located object was controlled for the axis between prime and target trials: the located object was positioned on the same axis in 16 prime and target trials and across axis in the other 16 trials per condition. The reference object and located object were positioned at the same, short distance from each other throughout the picture sequence.

In neutral pictures, the located object was placed along the vertical axis of the reference object leading to an alignment of the FORs. This alignment eliminated the need for the viewer to choose between the intrinsic and the relative FOR. For these configurations, eight additional objects were introduced (bench, box, chest, bottle, lamp, notebook, fish tank, carpet). Fifty distractor pictures were created that contained only a single discrete object in the foreground (pieces of furniture, toys, a book, a bottle, etc.).

The presentation of the visual stimuli was preceded by an auditorily presented sentence (in German) describing the object configuration and implicitly assigning the intrinsic or the relative FOR (or both in the control condition). The sentence “The *<located object>* is *<spatial term>* the *<reference object>*” was played over loudspeakers. See Figure 1 for an example picture of a scene with background with intrinsic and relative FOR. In the distractor trials the sentence “The *<object>* is *<adjective>*” was used, with a color or shape adjective. The picture remained on the screen until a response was given. There was no inter-trial interval.

Using a standard priming paradigm, we constructed three conditions of prime-target pairs: two experimental (match, mismatch) and one control condition. All three conditions had identical target trials in order to directly compare, both within-subject and within-item, the influence of the different prime conditions. Furthermore, each target trial was presented both with a relative and with an intrinsic FOR. In the match condition, prime and target trials used the same FOR (intrinsic-intrinsic, relative-relative). The mismatch condition contained different FORs for prime and target trial (intrinsic-relative, relative-intrinsic) and in the control condition a specific FOR was only used for the target picture (neutral-relative, neutral-intrinsic). We thus obtained a 2 × 3 design consisting of the factors “background” (with, without) and “priming condition” (match, mismatch, control) and accuracy and RTs of target trials as dependent variables. See Figure 2 for examples of Prime-Target pairs for each condition using a relative FOR in the Target trials.

With regard to scene type analysis, accuracy and RTs of prime trials were dependent variables in a 2 × 2 design with the factors “background” (with and without) and FOR (relative and intrinsic). Both FOR had identical prime trials to compare within-subject and within-item the effect of FOR processing.

To avoid effects resulting from simple repetition priming, we used different objects as well as different spatial terms for prime and target sentences. Furthermore, two distractor pictures were presented between successive prime-target pairs in order to avoid interactions between the FORs. In order to minimize other influences on FOR selection, we only used object configurations which did not show a functional relation between located objects and reference object (Carlson-Radvansky and Radvansky, 1996).

The randomization procedure took into account the priming condition, the rotation of the reference object (different rotations between prime-target pairs) and the reference object (changing objects between prime-target pairs) as well as the located object (position).

### Procedure

At the beginning of the experiment, the instructions were shown in written form on the monitor, informing the participants that they would hear a sentence after which a picture would be shown. The participants' task was to determine whether the sentence was an adequate description of the picture as quickly and accurately as possible and respond by pressing predefined yes/no keys on a button box. The experiment started with 5 practice trials followed by 400 experimental trials. In each trial, a sentence was presented acoustically (i.e., played on loudspeakers) while the computer monitor showed a white screen. Immediately afterwards, a picture of the aforementioned object configuration was shown. The picture remained on the screen until a response was given. Response times were measured from the onset of the picture display to the key-press response using E-Prime (Psychology Tools Software). The participants were unaware of the objective of the experiment and of the type of trials they were completing. No feedback was given during the experiment. The experiment lasted 30 min including a short break midway through.

## Results

Statistical analysis was carried out in “R” software (R Core Development Team, 2011) using the lme4 package (Bates et al., 2011). Linear mixed-effects models were used for the analysis of RTs and mixed-effects logistic regression (generalized linear mixed models, GLMM) for the analysis of accuracy.

RTs below 200 ms and above 4000 ms (1.4% of the data) were considered outliers, and were excluded from the analysis.

### Scene type

Descriptions regarding the neutral prime pictures were accepted in both conditions in 99% of the cases and were excluded from the analysis (33.3% of the data) as they did not require a choice between the intrinsic or the relative FOR. Accuracy and RTs of prime trials are presented in Table 1.

**Table 1:** **Accuracy and reaction times of prime trials**.

**FOR**	**Without background**		**With background**	
	**Accuracy (%)**	**RT in ms (*SD*)**	**Accuracy (%)**	**RT in ms (*SD*)**
Relative	47.6	1105.7 (597)	58.9	1146 (559)
Intrinsic	78	1148.2 (610)	55.4	1189 (561)
Control	99.1	749.8 (316)	99.4	802.1 (365)

In order to analyse effects of scene type and FOR on prime trial accuracy, we implemented a mixed-effects logistic regression analysis. We posited scene type, FOR and their interaction as fixed effects, and used random slopes and intercepts for subjects and items. We found a significant main effect of FOR, revealing a higher acceptability of the intrinsic compared to the relative FOR (β = −2.9, *SE* = 1.11, *Z* = −2.63, *p* < 0.001). Furthermore, there was a significant main effect of scene type (β = −2.14, *SE* = 0.72, *Z* = −2.99, *p* < 0.01) and a significant interaction between the two reflecting the higher accuracy of the intrinsic FOR in the condition without background (β = 2.92, *SE* = 1.48, *Z* = 1.97, *p* < 0.05).

RTs of the correct prime trials using the relative or intrinsic FOR were analysed (39.8% of the prime trials). Fitting a linear mixed-effects model with RT of the prime trial as dependent variable, a random slope and intercept for subjects and a random intercept for items, no significant main effects of background (β = −0.8912, *SE* = 84.3531, *t* = −0.011, *p* > 0.05) or FOR (β = −125.7638, *SE* = 91.61, *t* = 1.373, *p* > 0.05) were found.

### Priming effects

Subsets of data were used for the statistical analysis of priming effects, as we wish to consider only those trials in which the potential prime was accepted by the participants. In the analysis of the acceptability of target trials, we considered only trials that followed an accepted prime trial (72.9% of the trials). In the analysis of target RTs, we considered only trials in which both the prime and the target were accepted (45.9%).

**Figure 3:**
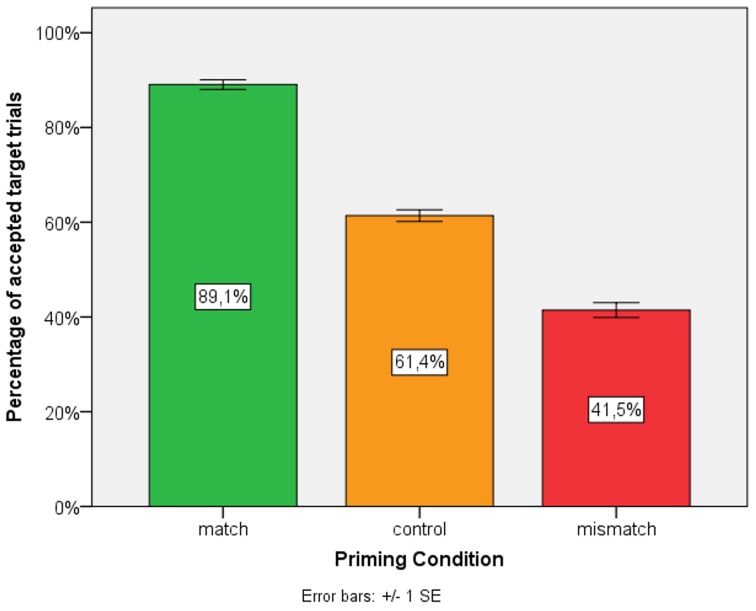
**Percentage of accepted target trials**.

For the analysis of target trial accuracy with regard to priming effects, we fitted a logistic mixed-effects model with scene type and priming condition as fixed effects, a random slope, and intercepts for subjects and a random intercept for items. Model comparison revealed a significant main effect of priming condition (*p* < 0.001) but not of scene type. Accuracy of match and mismatch condition differed significantly from the control condition revealing a higher accuracy in the match condition (β = 1, *SE* = 0.39, *Z* = 2.58, *p* < 0.01) and a lower accuracy in the mismatch condition (β = −0.8, *SE* = 0.37, *Z* = −2.14, *p* < 0.05). Figure 3 depicts the accuracy of target trials after an accurate prime trial collapsed across background version.

In order to analyse priming effects with regard to RTs, we fitted a linear mixed-effects model with full random slopes and intercepts for subjects and a random intercept for items. Taking the target RT as the dependent variable and the scene type and priming condition as independent variables, model comparison revealed that only the priming condition yielded a significant main effect (*p* < 0.05). This effect was attributable to the prolongation of RTs in the mismatch condition (β = 226.71, *SE* = 56.49, *t* = 4.01, *p* < 0.01) while the match condition did not differ significantly from the control condition (β = 42.92, *SE* = 53.00, *t* = 0.81, *p* > 0.05). Mean accuracy, RTs, and standard deviations (SD) are shown in Table 2. In order to quantify the priming effect, we calculated the differences in RTs by subtracting the mismatch and match from the control condition.

**Table 2:** **Mean reaction times and accuracy for target trials**.

**Primingcondition**	**Accuracy (%)**	**Reaction time in ms (SD)**	**Priming effect (ms)**
Match	89.1	1061 (515)	−44
Mismatch	41.5	1247 (662)	−230^*^
Control	61.4	1017 (516)	–

* p < 0.01.

## Discussion

### Influence of scene type on for processing

Our results revealed main effects of FOR and background on accuracy in the prime trials as well as a significant interaction between FOR and background. The interaction suggested that the clear preference for the intrinsic FOR in the condition without background was diminished in the condition with background, resulting in both FORs being accepted almost equally often. This equalization of accuracy resulted from a decrease in accuracy of the intrinsic FOR combined with an increase in accuracy of the relative FOR. The latter reflects our expectations that people are more likely to use the relative FOR and thus bring in their own perspective when the scene is more natural than in depictions without background elements. Being based on the viewer's direct perception (Miller and Johnson-Laird, 1976), Levinson (1996) claimed that “relative systems of spatial description build in a viewpoint” (p. 371), which implies that using the relative FOR demands an embodied viewer in order to establish this viewpoint. Requiring an embodied origin, the relative FOR can only be processed in depictions of scenes via a mental simulation of the viewer in the scene. This stands in line with Wilson's (2002) idea that off-line cognition is body based and that sensorimotor resources are used to simulate physical aspects of the world. Thus, when natural elements are included, this increases the probability of establishing a ternary relationship resulting in a “boost” in the availability of the relative FOR.

Interpreting the decrease in acceptability of the intrinsic FOR in the scene with background, as indicated by the main effect of background condition, however, is not straightforward. General preferences for the intrinsic FOR have been reported in previous studies (e.g., Carlson-Radvansky and Irwin, 1993; Carlson-Radvansky and Radvansky, 1996; Taylor et al., 1999; Taylor and Rapp, 2004). Given that these studies did not use scenes with background, the results are comparable to our findings for the scene without background. The scene with background may, however, have led to a decrease in this preference by reducing its saliency and increasing the saliency of the relative FOR. Findings that point to the influence of the environment on FOR selection have been described by Taylor and Tversky (1996), who showed that participants used relative, intrinsic, and extrinsic frames of reference differently depending on the environment they were asked to describe. They interpreted their findings as a reflection of how we interact with the environment.

Another line of research that points in this direction are findings from studies using neuroimaging technology to investigate brain activation patterns resulting from different visual stimuli. In general, stimuli embedded in a scene and stimuli presented without background scene induce different brain activation patterns. Using fMRI technology, a certain brain region, the “parahippocampal place area” (PPA), could be identified, which responded selectively to scenes but not to single objects or object arrays (e.g., Epstein and Kanwisher, 1998; Henderson et al., 2008). In addition, it has been reported that the visually perceived spatial structure of the environment is processed by the PPA (Epstein et al., 1999) and that the PPA is viewpoint-specific and thus plays a crucial role in establishing the relationship between the viewer and the spatial structures of the environment (Epstein et al., 2003). Thus, these findings may reflect that the relative FOR, for which the establishment of a relationship between the viewer and spatial structures is a prerequisite, is more likely to be used in scenes compared to object arrays.

Interestingly, activation of brain areas in the middle temporal and middle superior temporal areas have recently been reported as resulting from mentally simulated motion in the processing of static pictures (Coventry et al., in press). The localisatory differences may be explained by the fact that mental simulation did not require a viewpoint in the scene and the stimuli were pictures without background.

Our results indicate that humans have different preferences for FORs depending on the scene type. Following this idea, we assume a further decrease in preference for the intrinsic FOR in favor of the relative FOR when participants are embodied in the scene. This is a matter for further investigations.

### Priming effects

Our experiment was designed to investigate priming effects for RTs and accuracy ratings. The results showed longer RTs and lower accuracy ratings for different FORs, but for same FORS we only found higher accuracy ratings and no RT effect.

Longer RTs for different FORs have been reported previously (Carlson-Radvansky and Jiang, 1998; Carlson and van Deman, 2008) and our results support these earlier findings. The prolongation of RTs in trials that required a switching of FORs has been interpreted as inhibition (Carlson-Radvansky and Jiang, 1998; Carlson and van Deman, 2008). This inhibition increases the cognitive effort needed for the adoption of different FORs in subsequent trials.

However, we did not only find longer RTs but also lower accuracy ratings. We interpret this as resulting from the strength of inhibition. Longer RTs reflect relatively mild inhibition, as the FOR that was inhibited could still be adopted. The fact that a large proportion of trials in the mismatch condition were rejected reflects a more powerful inhibition, one that made the FOR completely unavailable.

With regard to the RTs, we found no processing advantages in the match condition. However, the accuracy of target trials in the match condition was significantly higher than in the control condition. This suggests the presence of a priming effect. It has been claimed that, initially, multiple FORs are active and compete for selection (Carlson-Radvansky and Irwin, 1994; Carlson-Radvansky and Jiang, 1998). Our results reveal that the selection of a specific FOR leads to a persistently higher level of activation in the subsequent trial and thus to a selection advantage. This indicates that FOR selection is not only accompanied by inhibition of the non-selected FOR but also by a higher level of activation of the selected FOR.

The finding that participants showed a corresponding effect for accuracy in both conditions, but for RT there was only a prolongation in the mismatch condition, is difficult to explain. We speculate that the higher processing complexity of switching FOR in the mismatch condition also leads to a higher error rate in the sentence verification, whereas the easier processing in the match condition makes the sentence verification less error prone. This would imply that there is no speed-accuracy trade-off in this task, which is supported by an inspection of the RTs in the trials with erroneous responses: the erroneous responses were *slower* (*M* = 1184, *SD* = 535) than the correct responses (*M* = 1139, *SD* = 577, *t*_(2559)_ = 2, *p* < 0.05).

The preference for using the same FOR has also been shown in a dialogue study, in which speakers tended to use the same FOR as their interlocutor had (Watson et al., 2004). This alignment was attributed to priming effects and has been described for different levels of linguistic representation including abstract concepts such as FORs (see Pickering and Garrod, 2004, for an overview). Priming effects are thus discussed to play a central role in communication (Pickering and Garrod, 2004).

In conclusion, our results show that people are more likely to describe a scene from an egocentric point of view when the scene has a realistic background. We explain this phenomenon by assuming that the presence of a background stimulates an embodied mental simulation of a real scene.

More generally, our results show that priming does not only have facilitatory effects on referential communication, but can also slow us down or decrease our communicational efficiency, depending on the sequential context in which utterances occur.

### Conflict of interest statement

The authors declare that the research was conducted in the absence of any commercial or financial relationships that could be construed as a potential conflict of interest.

## Footnotes

^1^This effect has also been called “negative priming” in the literature (Carlson-Radvansky and Jiang, 1998; Carlson and van Deman, 2008).
